# Implications of a New Generation of Tissue Expanders for Post-Mastectomy Radiotherapy in Breast Reconstruction: A Retrospective Single-Center Study

**DOI:** 10.3390/jcm15114224

**Published:** 2026-05-29

**Authors:** Glenda Giorgia Caputo, Anna Scarabosio, Gaetano Pisano, Carmen Giunco, Agnese Prisco, Eugenia Moretti

**Affiliations:** 1 Plastic and Reconstructive Surgery Department, Azienda Sanitaria Universitaria Friuli Centrale, 33100 Udine, Italy; scarabosioanna@gmail.com (A.S.); pisanogaetano95@gmail.com (G.P.); 2 Department of Medical Physics, Azienda Sanitaria Universitaria Friuli Centrale, 33100 Udine, Italy; carmen.giunco@asufc.sanita.fvg.it (C.G.); eugenia.moretti@asufc.sanita.fvg.it (E.M.); 3 Department of Radiation Oncology, Azienda Sanitaria Universitaria Friuli Centrale, 33100 Udine, Italy; agnese.prisco@asufc.sanita.fvg.it

**Keywords:** non-metallic valve expander, RFID valve, post-mastectomy radiotherapy, two-stage reconstruction, Motiva Flora

## Abstract

**Background:** Tissue expanders with metallic ports are commonly used in post-mastectomy breast reconstruction but can produce significant computed tomography (CT) artifacts, which impair accurate delineation of target volumes during radiotherapy planning. The Motiva Flora^®^ expander incorporates an integrated radiofrequency identification (RFID) valve, designed to be magnet-free and magnetic resonance imaging (MRI)-conditional, potentially minimizing image distortion and improving the precision of treatment planning. This pilot study aims to quantitatively compare the extent of CT image distortion observed in radiotherapy simulation scans between conventional metallic-valve expanders and RFID-valve expanders, evaluating their impact on radiotherapy planning quality. **Methods:** Between January 2024 and September 2025, fourteen consecutive patients who underwent post-mastectomy two-stage breast reconstruction followed by adjuvant RT at Hospital Santa Maria della Misericordia (Udine, Italy) were included. Seven patients received Motiva Flora^®^ tissue expanders with a non-metallic RFID port, and seven received Mentor CPX4^®^ expanders with a conventional metallic port. The volume of areas with a significant level of artifacts (artifact volume) was quantitatively evaluated by delineating the CT image area of distortion caused by the valve. Moreover, a comparison of the ratio between artifact volume and clinical target volume (artifact volume/CTV volume) between expander types to assess potential imaging-related distortions has been made. Group comparisons of volume ratio were performed using Welch’s *t*-test. **Results:** Patients reconstructed with Motiva Flora^®^ showed a mean artifact volume of 24.5 ± 10.3 cc, whereas those with Mentor CPX4^®^ expanders presented a mean artifact volume of 64.2 ± 38.1 cc. The ratio between artifact volume and clinical target volume (CTV) was lower in patients reconstructed with Motiva expanders compared to those reconstructed with Mentor expanders and this difference was significant with Welch’s *t*-test (*p* = 0.046). **Conclusions:** The reduced CT distortion observed with the RFID valve-equipped Motiva Flora suggests a superior radiological compatibility compared to conventional metallic-port expanders, with potential to enhance the accuracy of radiotherapy planning.

## 1. Introduction

Post-mastectomy breast reconstruction is a fundamental component of comprehensive breast cancer care, providing both aesthetic restoration and well-documented psychological benefits [1]. Beyond the restoration of body image, breast reconstruction has been shown to significantly improve quality of life, self-esteem, and social reintegration, making it an integral part of the oncologic treatment pathway rather than a purely reconstructive option. Alongside immediate direct-to-implant (DTI) reconstruction and autologous flap, the two-stage reconstructive approach remains widely adopted. This approach continues to represent a versatile and reliable strategy, particularly in complex oncologic scenarios, allowing surgeons to optimize soft tissue conditions before definitive reconstruction. This strategy involves placement of a temporary tissue expander, either in a submuscular or prepectoral plane, followed by a second-stage procedure to replace the expander with a permanent implant or an autologous flap [2]. The choice between submuscular and prepectoral positioning is typically influenced by patient-specific factors, including mastectomy flap thickness, oncologic considerations, and surgeon preference, with increasing adoption of prepectoral techniques in recent years due to improved biomaterials and reduced animation deformity.

In patients requiring post-mastectomy radiation therapy (PMRT), the two-stage approach provides additional advantages. Radiotherapy remains a cornerstone in reducing local recurrence rates in high-risk breast cancer patients; however, it is also associated with well-known adverse effects on reconstructed tissues, including fibrosis, capsular contracture, and impaired wound healing. Delivering radiation therapy to a temporary expander rather than a definitive implant allows clinician to assess tissue response prior to final reconstruction, potentially guiding a shift toward autologous reconstruction, which remains the gold standard in irradiated patients. This staged strategy therefore offers a degree of flexibility, enabling individualized reconstructive planning based on the biological response to radiation and minimizing the risk of long-term complications.

However, conventional tissue expanders frequently contain metallic components, most commonly titanium or stainless-steel ports, which generate significant computed tomography (CT) artifacts that degrade image quality that may interfere with target delineation and in general, impact radiotherapy planning accuracy [2,3,4]. These artifacts typically manifest as streaking and beam hardening effects, which can obscure adjacent anatomical structures and compromise the visualization of critical organs at risk. In particular, Hounsfield Unit (HU) distortion caused by metallic ports introduces uncertainties in dose calculation in the region around the device, affecting its consistency. Such inaccuracies may lead to suboptimal dose distribution, with potential risks of underdosing the target volume or overdosing surrounding healthy tissues, ultimately impacting treatment efficacy and safety.

To address these limitations, the Motiva Flora^®^ expander was developed with a non-metallic RFID (radiofrequency identification) port designed to reduce imaging artifacts and improve CT-based radiotherapy planning [5,6]. By eliminating metallic components, this device aims to provide more homogeneous imaging characteristics, facilitating more accurate contouring and dose calculation. Furthermore, the integration of RFID technology allows non-invasive identification and localization of the port, potentially improving intraoperative and postoperative management without compromising imaging quality [7].

The aim of the present study was to quantitatively compare CT image distortion between Motiva Flora^®^ expanders and traditional metallic-port expanders in patients undergoing PMRT, evaluating their impact on artifact volume and target delineation. We hypothesized that the use of a non-metallic port would significantly reduce artifact burden and improve radiotherapy planning precision, thereby supporting its adoption in patients requiring adjuvant irradiation.

## 2. Materials and Methods

### 2.1. Study Design and Patient Selection

This retrospective study assessed the impact of two different types of breast tissue expander, both with an integrated valve on the anterior surface, on CT image quality in the setting of adjuvant radiation therapy (RT).

Between January 2024 and September 2025, fourteen consecutive patients who underwent post-mastectomy two-stage breast reconstruction followed by adjuvant RT at Hospital Santa Maria della Misericordia—ASUFC (Udine, Italy) were included.

*Inclusion criteria* were:

- Female patients undergoing immediate two-stage implant-based breast reconstruction following mastectomy;
- Use of either Motiva Flora^®^ or Mentor CPX4^®^ tissue expanders;
- Planned postoperative adjuvant radiotherapy;
- Availability of complete CT simulation imaging data for radiotherapy planning.

*Exclusion criteria* were:

- Previous chest wall irradiation;
- Incomplete radiological imaging data;
- Patients who did not complete radiotherapy treatment;
- Patients lost to follow-up during the expansion period.

Seven patients received Motiva Flora^®^ tissue expanders (Establishment Labs S.A., Alajuela, Costa Rica) with a non-metallic RFID port, and seven received Mentor CPX4^®^ expanders (Mentor Worldwide LLC, Irvine, CA, USA) with a conventional metallic port. CT simulation data were available for all cases. Patients with previous chest wall irradiation or incomplete imaging were excluded.

### 2.2. Surgical Technique and Radiotherapy Procedure

At the time of mastectomy, a submuscular tissue expander was placed beneath the pectoralis major muscle. The muscle was elevated from the chest wall to create a submuscular pocket, and the inferior border of the pectoralis major was sutured to an acellular dermal matrix (ADM), following a dual-plane reconstruction technique. Expansion began three weeks postoperatively and continued until the desired volume was achieved; it was interrupted from when the patient underwent the CT scan to plan radiotherapy to the end of radiotherapy. If further volume adjustments were necessary, inflations were resumed at the end of radiotherapy, when local conditions allowed. The final expansion volume was maintained for at least six months to allow tissue stabilization prior to the second-stage reconstruction. The definitive reconstructive procedure consisted of either replacement of the expander with a permanent implant or conversion to autologous reconstruction. Radiotherapy planning was performed using CT-based simulation according to institutional protocols. Target volumes and organs at risk were delineated using AI-assisted contouring tools, followed by manual review and validation by experienced radiation oncologists. Treatment plans were calculated by medical physicists using advanced dose calculation algorithms to optimize target coverage while minimizing exposure to surrounding healthy tissues.

### 2.3. Evaluation Parameter and Statistical Analysis

**Figure 1:**
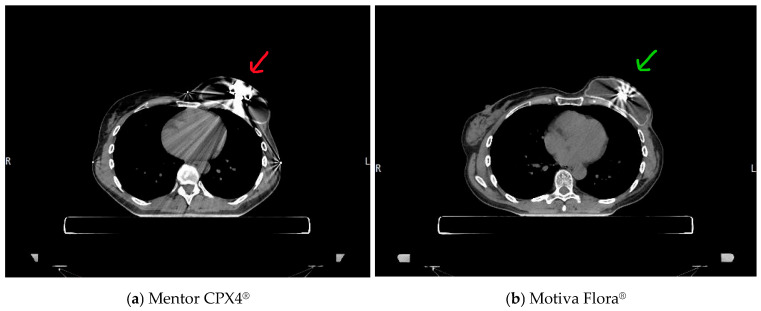
Representative axial CT images showing artifact generation from tissue expanders: (**a**) Mentor CPX4^®^ expander with metallic port, demonstrating pronounced beam-hardening artifacts; (**b**) Motiva Flora^®^ expander with non-metallic RFID port, showing reduced artifact burden.

CT artifacts were delineated as the apparent low-density volume surrounding the expander port, expressed in cubic centimeters (cc). The results of the segmentation process were independently verified by two medical physicists with high expertise in breast radiotherapy planning. The primary endpoint of the current work was the analysis of the quality of planning imaging with the two generations of submuscular tissue expanders. The volume of areas with a significant level of artifacts (artifact volume) was quantitatively evaluated by delineating the CT image area of distortion caused by the valve. Secondary endpoints included a comparison of the ratio between artifact volume and clinical target volume (artifact volume/CTV volume) between expander types to assess potential imaging-related distortions (Figure 1). Differences in volume ratio between the 2 groups of expanders were evaluated using Welch’s *t*-test, which was chosen due to unequal variances and small sample size. Given the exploratory and pilot nature of the present study, a formal a priori power analysis was not performed. The current investigation was designed as a preliminary proof-of-concept study aimed at evaluating potential differences in CT imaging artifacts between the two expander types and generating hypothesis-driven data for future larger prospective investigations.

## 3. Results

A total of 14 patients were included in the analysis: seven reconstructed with Motiva Flora^®^ expanders and seven with Mentor CPX4^®^ expanders. In Table 1, the quantitative measurements are reported for each patient.

Following radiation therapy, six patients underwent delayed autologous reconstruction with a DIEP flap, one patient underwent implant-based reconstruction, and five patients are currently awaiting definitive reconstructive decisions. Two expanders were removed (one for infection and one for capsular contracture) and there is a pending decision on five patients. Median post-radiation therapy follow-up was 12.3 months.

### Quantitative Analysis

The mean clinical target volume (CTV) was 593.5 ± 240.8 cc for Motiva Flora^®^ and 607.7 ± 211.2 cc for Mentor CPX4^®^ expanders. 

**Figure 2:**
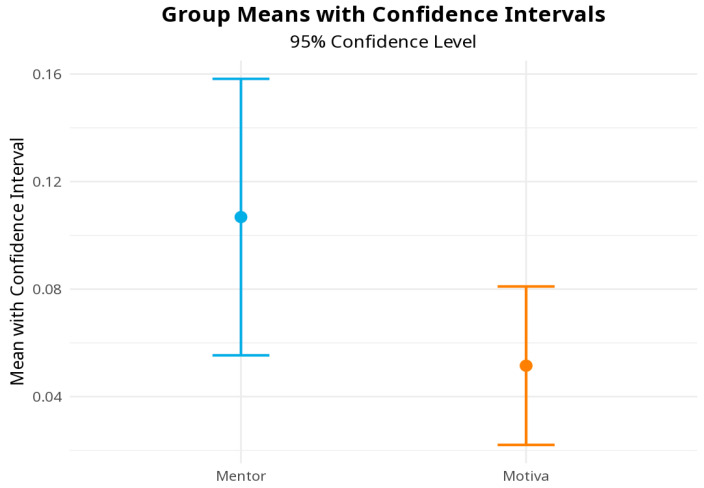
Comparison of the mean artifact-to-CTV ratio between Mentor CPX4^®^ expanders and Motiva Flora^®^.

Patients reconstructed with Motiva Flora^®^ showed a mean artifact volume of 24.5 ± 10.3 cc (range 4.4–38.3 cc), whereas those with Mentor CPX4^®^ expanders presented a mean artifact volume of 64.2 ± 38.1 cc (range 13.8–120.4 cc). We analyzed the ratio between artifact volume and clinical target volume (CTV). It was lower in patients reconstructed with Motiva expanders compared to those reconstructed with Mentor expanders. The mean artifact-to-CTV ratio was 0.051 (SD = 0.0319) for Motiva expanders and 0.107 (SD = 0.0556) for Mentor expanders. Welch’s *t*-test revealed a statistically significant difference between the group means (*p* = 0.046, Figure 2).

## 4. Discussion

In this study, we demonstrate that non-metallic RFID-port tissue expanders reduce CT-simulation imaging artifacts. These findings reinforce the concept that expander design—specifically port composition—plays a critical role in imaging fidelity and radiotherapy planning accuracy in reconstructed breasts. Importantly, our results highlight how seemingly minor device-related characteristics can have clinically meaningful downstream effects on oncologic treatment planning, underscoring the need for closer integration between reconstructive surgery and radiation oncology [1]. From a radiotherapy perspective, metallic ports composed of titanium or stainless steel are well known to induce beam-hardening effects and Hounsfield Unit (HU) distortion, which can propagate beyond the immediate vicinity of the implant and compromise image quality across the chest wall. These artifacts degrade image quality and can make it difficult to delineate the true anatomical interface between the expander, pectoralis muscle, ribs, and adjacent organs at risk, such as the heart and lungs, particularly in left-sided breast cancers. This issue is particularly relevant in contemporary radiotherapy, where increasing emphasis is placed on highly conformal techniques and precise dose sculpting around critical structures [8]. Even when modern treatment planning systems incorporate artifact-reduction algorithms or density overrides, residual uncertainties often persist, potentially affecting dose calculation accuracy and contouring reproducibility [2,9,10,11,12]. Such uncertainties may introduce interobserver variability and reduce the robustness of treatment planning, particularly in neighboring anatomical regions. The non-metallic RFID port of the Motiva Flora^®^ expander significantly mitigates these limitations by reducing density heterogeneity within the reconstructed breast. The markedly smaller artifact volume observed in our cohort translated into clearer visualization of soft tissues and bony landmarks, facilitating more confident delineation of clinical target volumes. This improved visualization may also enhance consistency across planning sessions and between operators, an aspect that is increasingly recognized as critical in modern radiotherapy workflows.

In our cohort, Motiva Flora^®^ expanders exhibited a lower artifact-to-CTV ratio compared to Mentor expanders, indicating a reduced relative artifact burden with respect to the target volume. This reduction translated into clearer visualization of the surrounding soft tissue, facilitating more confident delineation of clinical target volumes. The use of a normalized metric such as the artifact-to-CTV ratio further strengthens the clinical relevance of our findings, as it contextualizes artifact burden in relation to the actual treatment volume rather than as an isolated parameter.

Beyond target definition, artifact reduction carries important dosimetric implications. Beam-hardening artifacts can alter HU values used for electron density mapping, potentially leading to local dose perturbations, especially in intensity-modulated radiotherapy (IMRT) techniques. Inaccurate electron density assignment may result in clinically relevant deviations in dose distribution, particularly in regions where steep dose gradients are required. The relevance of expander-related artifacts becomes even more pronounced in the context of emerging radiotherapy modalities. In proton therapy, metallic components can cause substantial range uncertainties due to their high density and sharp interfaces, resulting in unpredictable dose deposition and potential compromise of target coverage or increased exposure to organs at risk. As proton therapy becomes more widely adopted for selected breast cancer patients, especially those with left-sided disease or high cardiac risk, the use of low-density, non-metallic expanders may represent an enabling factor for safe and effective treatment delivery [13]. In this context, expander design may evolve from a purely reconstructive consideration to a determining factor in selecting optimal radiotherapy modalities.

From a reconstructive standpoint, the two-stage approach in patients requiring PMRT offers strategic flexibility. Delivering radiotherapy to a temporary expander rather than a definitive implant allows surgeons to evaluate tissue tolerance to irradiation before committing to a final reconstructive modality. This adaptability is particularly valuable in patients with unpredictable soft-tissue responses, where delayed decision-making can optimize both aesthetic and functional outcomes.

At our institution, PMRT is integrated into a multidisciplinary treatment algorithm involving Plastic Surgery, Radiation Oncology, and Medical Physics. All patients in this series underwent submuscular expander placement, reflecting our preference for enhanced soft-tissue coverage and protection of the implant in the irradiated field. This approach also provides an additional layer between the expander and the skin envelope, which may be advantageous in mitigating radiation-induced complications. While prepectoral reconstruction has gained increasing popularity and has shown promising results in carefully selected patients, concerns persist regarding higher rates of seroma formation, wound complications, and implant loss following PMRT. These concerns are amplified in irradiated fields, where compromised vascularity and tissue quality may exacerbate complication profiles. In irradiated settings, submuscular reconstruction remains a conservative and reproducible option that aligns well with radiotherapy requirements [14,15]. Autologous reconstruction continues to represent the gold standard for patients undergoing PMRT. In our cohort, most patients ultimately underwent delayed DIEP flap reconstruction, consistent with extensive evidence demonstrating lower long-term complication rates, improved durability, and superior patient-reported outcomes compared with implant-based reconstruction in irradiated breasts [16,17]. This preference reflects a broader trend toward delayed autologous reconstruction in irradiated patients, aiming to minimize the deleterious effects of radiation on final reconstructive outcomes [18]. The use of vascularized autologous tissue provides robust coverage of the chest wall, mitigates radiation-induced fibrosis, and reduces the risk of capsular contracture or reconstructive failure [19]. Nevertheless, patient preference, comorbidities, and oncologic factors necessitate individualized reconstructive planning, and implant-based reconstruction may still be appropriate in selected cases [20]. 

Improved pre-treatment CT imaging quality and thus, more precise target and organ-at-risk delineation could limit unnecessary radiation exposure to surrounding healthy tissues, including the internal mammary vessels, which are critical recipient vessels for subsequent autologous breast reconstruction. Preservation of these vessels may be particularly relevant in patients scheduled for autologous reconstruction, in which radiation-induced vascular damage can negatively affect surgical outcomes. This aspect further reinforces the importance of considering future reconstructive steps during initial radiotherapy planning, highlighting the longitudinal nature of breast cancer care.

This study has several limitations that should be carefully acknowledged. First, the retrospective design inherently exposes the analysis to potential selection and observational biases. In addition, the study population was relatively small, consisting of only 14 patients, and no formal a priori power analysis was performed due to the exploratory and pilot nature of the investigation. Consequently, the statistical findings should be interpreted with caution, as the limited sample size may reduce the robustness and reproducibility of the observed associations.

Although a statistically significant difference in artifact burden between the two expander types was identified, the present cohort may not adequately capture the full variability encountered in clinical practice. Factors such as patient anatomy, body habitus, expander positioning, expansion volume, and radiotherapy planning techniques could all potentially influence imaging artifacts and dosimetric characteristics. With a limited number of patients, these confounding variables could not be fully controlled or stratified.

Furthermore, the absence of a formal power calculation increases the risk of both type I and type II statistical errors. Therefore, the present findings should primarily be considered hypothesis-generating rather than definitive evidence supporting superiority of one device over another. Nevertheless, despite the small cohort, the magnitude and consistency of artifact reduction observed with the non-metallic RFID-port expanders suggest a potentially meaningful clinical effect that warrants further investigation.

Another important limitation is the absence of detailed dosimetric analyses and long-term oncologic or reconstructive outcome evaluation. While reduced CT artifact burden likely improves contouring accuracy and radiotherapy planning confidence, the present study was not designed to determine whether these imaging improvements directly translate into clinically measurable differences in radiation delivery, toxicity profiles, reconstructive complications, or oncologic outcomes.

Future prospective multicenter studies with larger patient populations, standardized radiotherapy protocols, and formal dosimetric analyses will therefore be essential to validate these preliminary findings and to better define the clinical role of non-metallic tissue expanders in patients undergoing post-mastectomy radiotherapy.

Despite these limitations, our findings provide clinically relevant evidence supporting the use of non-metallic RFID port expanders in patients undergoing PMRT. By significantly improving CT image quality without altering target volumes, this technology enhances radiotherapy planning precision while preserving reconstructive flexibility. Ultimately, the adoption of imaging-compatible reconstructive devices may contribute to a paradigm shift toward more integrated, patient-specific treatment pathways. As breast cancer treatment continues to evolve toward increasingly personalized, multidisciplinary care, the integration of reconstructive devices optimized for radiotherapy represents a meaningful step toward improving both oncologic safety and reconstructive outcomes.

## 5. Conclusions

The present study demonstrates that non-metallic RFID-port tissue expanders are associated with a significant reduction in CT-simulation imaging artifacts in patients undergoing post-mastectomy radiotherapy. Improved image quality allows clearer visualization of anatomical structures, facilitating more accurate delineation of clinical target volumes and adjacent organs at risk. By minimizing density heterogeneity and Hounsfield Unit distortion, non-metallic expanders may improve the reliability and reproducibility of radiotherapy planning compared with conventional metallic-port devices.

These findings may also have relevant clinical implications. Improved target definition and reduced uncertainty in dose calculation could contribute to more precise radiation delivery while limiting unintended exposure to surrounding healthy tissues such as the heart, lungs, and internal mammary vessels. This may be particularly important in left-sided breast cancer and in patients undergoing advanced radiotherapy techniques, including intensity-modulated radiotherapy and proton therapy.

From a reconstructive perspective, imaging-compatible expanders further support a multidisciplinary approach to breast cancer care by integrating reconstructive and oncologic needs. In addition, more accurate radiation delivery may help preserve soft tissue quality and recipient vessels in patients undergoing delayed autologous reconstruction.

Although limited by its retrospective design and relatively small sample size, this study provides preliminary quantitative evidence supporting the clinical utility of non-metallic RFID-port expanders in the setting of post-mastectomy radiotherapy. Future prospective studies with larger cohorts and detailed dosimetric analyses are needed to further validate these findings.

## Figures and Tables

**Table 1:** Individual patient data including expander type, CT artifact volume (cc), clinical target volume (CTV, cc), and artifact-to-CTV ratio.

Patient ID	Type	Artifact Expander (cc)	CTV (cc)	Artifact Expander/CTV
1	Motiva	23.4	910.9	0.0257
2	Motiva	23.3	602.3	0.0387
3	Motiva	23.8	553.0	0.0430
4	Motiva	29.7	351.9	0.0844
5	Motiva	4.4	928.8	0.0047
6	Motiva	38.3	440.3	0.0870
7	Motiva	28.3	367.1	0.0771
8	Mentor	77.2	484.1	0.1595
9	Mentor	104.3	811.7	0.1285
10	Mentor	120.4	665.1	0.1810
11	Mentor	50.1	580.0	0.0864
12	Mentor	13.8	387.7	0.0356
13	Mentor	44.4	386.5	0.1149
14	Mentor	39.1	938.5	0.0417

## Data Availability

The data presented in this study are available from the corresponding author on reasonable request.

## Abbreviations

The following abbreviations are used in this manuscript:

ADM	Acellular Dermal Matrix
CT	Computed Tomography
CTV	Clinical Target Volume
DIEP	Deep Inferior Epigastric Perforator
DTI	Direct-to-implant
HU	Hounsfield Unit
IMRT	Intensity-modulated Radiotherapy
MRI	Magnetic Resonance Imaging
PMRT	Post-mastectomy Radiotherapy
RFID	Radiofrequency Identification
RT	Radiotherapy
